# Solid-Phase Microextraction Techniques and Application in Food and Horticultural Crops

**DOI:** 10.3390/molecules28196880

**Published:** 2023-09-29

**Authors:** Snezana Agatonovic-Kustrin, Vladimir Gegechkori, Tamara Kobakhidze, David Morton

**Affiliations:** 1 Department of Pharmaceutical and Toxicological Chemistry Named after Arzamastsev of the Institute of Pharmacy, I.M. Sechenov First Moscow State Medical University (Sechenov University), 119991 Moscow, Russia; vgegechkori@gmail.com (V.G.); kobakhidze_t_i@staff.sechenov.ru (T.K.); d.morton@latrobe.edu.au (D.M.); 2 School of Rural Clinical Sciences, La Trobe Institute for Molecular Sciences, La Trobe University, Edwards Rd, Bendigo 3550, Australia

**Keywords:** foods, head space extraction, gas chromatography–mass spectroscopy, pesticides, foods, solid phase microextraction, volatile compounds

## Abstract

Solid-phase microextraction (SPME) is a sample preparation technique which utilizes small amounts of an extraction phase for the extraction of target analytes from investigated sample matrices. Its simplicity of use, relatively short sample processing time, and fiber reusability have made SPME an attractive choice for many analytical applications. SPME has been widely applied to the sampling and analysis of environmental, food, aromatic, metallic, forensic, and pharmaceutical samples. Solid phase microextraction is used in horticultural crops, for example, to determine water and soil contaminants (pesticides, alcohols, phenols, amines, herbicides, etc.). SPME is also used in the food industry to separate biologically active substances in food products for various purposes, for example, disease prevention, determining the smell of food products, and analyzing tastes. SPME has been applied to forensic analysis to determine the alcohol concentration in blood and that of sugar in urine. This method has also been widely used in pharmaceutical analysis. It is a solvent-free sample preparation technique that integrates sampling, isolation, and concentration. This review focuses on recent work on the use of SPME techniques in the analysis of food and horticultural crops.

## 1. Introduction

The composition of food is incredibly diverse and complex, containing proteins, carbohydrates, minerals, vitamins, etc. Such complex compositions prompt the need for advanced methods in quality and safety evaluation. For example, most food products have a defined shelf-life. Thus, knowing the original and modified chromatographic patterns of fresh and processed/stored food products is essential in determining the compositional changes that food products may undergo over the time, such as microbial activity or the leaching of chemicals from packaging materials. In addition, the analysis of pesticides is a primary focus in the safety evaluation of food products. Ideally, a method for pesticide analysis in food should be rapid and easy to run with a minimum use of solvents, while providing a certain degree of selectivity and covering a wide scope of analyte–matrix combinations [1]. Additionally, more automated methods of analysis have the advantage of high sample throughput and minimize errors associated with human error [2]. Solid-phase microextraction (SPME) is able to meet most of these requirements via the integration of sampling, extraction, concentration, and sample introduction into a single solvent-free step. It was first developed and initially reported in the late 1980s/early 1990s by Pawliszyn and co-workers [3,4,5]. It has since been developed as a sample preparation tool for a wide range of applications, such as food adulteration analysis, water analysis, food analysis, wine and beer analysis, pharmaceutical and biomedical analysis, soil analysis, etc. [6,7,8,9,10,11,12,13]. The aim of this review is to focus on recent work that reports on the use of SMPE techniques in the analysis of food and horticultural crops.

The major advantages of SPME include its easy miniaturization, automation of the analysis procedure, and convenience in coupling with gas chromatography (GC), high-performance liquid chromatography (HPLC) or capillary electrophoresis (CE). Due to the complexity of food matrices and the low concentration of the analytes analyzed, traditional sample preparation methods involve multiple sample pre-treatment steps; that is, the isolation/extraction of the components of interest from the sample matrix, purification, and then pre-concentration in order to reach sufficient sensitivity for a particular analytical method. Once the sample is prepared, the analysis is then run on the instrument of choice, such as a GC or a HPLC.

In contrast to commonly used liquid–liquid extraction (LLE) or solid-phase extraction (SPE) sample preparation methods, SPME involves just two distinct analyte transfer steps: (1) the adsorption/extraction of the analyte into a layer of adsorptive material usually coated on a fused silica fiber, followed by (2) the transfer (desorption) of the adsorbed analytes from the fiber into the inlet of a chromatography column [14]. Extraction (on the SPME fiber) is carried out either via the direct immersion (DI) of the fiber into a liquid sample, or via exposing the fiber to the sample vapor phase containing volatile analytes (headspace extraction (HS)). Analytes then adsorb, and accumulate on the coated fiber. After a certain period of time, the analyte concentration in the SPME layer will reach an equilibrium level. The fiber is then removed and the sample is injected into a gas chromatograph via thermal desorption from the fiber (i.e., heating the fiber). For liquid chromatography, the desorption of analytes into a solvent occurs before the injection of the resultant solution into a HPLC column. Not only does the use of SMPE provide a simpler sample preparation process, decreasing the time required to process samples, but it should also lead to a reduction in errors associated with the procedure, as statistically, the amount of uncertainty in a method is directly related to the number of steps it contains.

**Figure 1:**
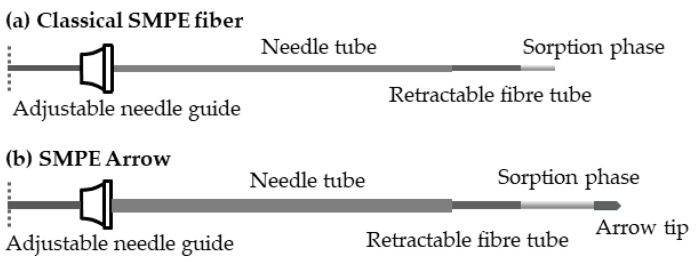
Schematic diagram of (**a**) a classical SMPE coated fiber, and (**b**) a SPME Arrow coated rod.

A SPME configuration which enables larger samples to be collected from a sample matrix is the prep and load solid-phase microextraction (PAL SPME) Arrow. This consists of a stainless steel rod onto which the sorbent is coated, and is capped with a conical tip, which facilitates penetration through a septum. The diameter and length of the rod is much larger than that of a a normal SPME fiber, enabling a much larger amount of sorbent to be used, for example, a 1 cm × 100 μm (0.6 μL) classical SMPE fiber compared to a 3 cm × 250 μm (15.3 μL) SPME Arrow (Figure 1) [15]. However, in order to extract a larger amount of the sample, adsorption and desorption times will be longer.

A SPME fiber normally has a length of 1 cm and consists of a solid fused silica substrate coated with a suitable polymeric stationary extraction phase, such as divinylbenzene (DVB), polyacrylate (PA), polydimethylsiloxane (PDMS), Carboxen (CAR), Carbowax (CW), and templated resin (TPR). These are used either individually or as a mixture of two to three components [16,17,18].

Despite the advantages in using SPME for sample preparation, analyses of complex matrices such as food create significant challenges, especially when developing methods for analysis for analytes present at very low concentrations. In the past, its use in food analysis has been limited by the number of commercially available sorbent coatings, their short lifetime, sensitivity and matrix effect. Commercially available SPME fibers are fragile with a relatively low operating temperature due to the thermal instability of the sorbent coating [19]. Furthermore, the direct immersion of the extraction phase in a complex sample matrix can cause damage to the extraction phase, due to the irreversible adsorption of macromolecules from the sample matrix that will shorten the coating lifetime and change the method’s sensitivity or the extraction properties of the coating (matrix effect). Potential swelling in common organic liquid chromatography solvents may also cause matrix effects [19]. Stir bar sorptive extraction (SBSE) can also be used when larger samples need to be extracted from a sample matrix. This involves the use of a magnetic stirrer bar with a sorbent coating to extract analytes from a sample matrix [20]. This also has the advantage of not requiring an extraction solvent but results in more labor-intensive analysis when compared to that in SPME, which can automated.

The extent of the adsorption of analytes onto a SPME fiber is dependent on temperature, and on the physicochemical properties of the analyte and the stationary phase on the fiber. This is quantified via the distribution coefficient (*K*_fs_) between the two phases where,

*K*_fs_ = [A]_f_/[A]_s_ (1)

*K*_fs_ gives a measure of the extent of the adsorption of analytes onto the fiber, where [A]_f_ and [A]_s_ are the equilibrium concentrations of the analyte in the fiber and sample phases, respectively. Note that the actual amount of analyte extracted is dependent on both *K*_fs_ and the amounts of stationary and sample phases present [14]. Generally, a polar fiber coating will more effectively absorb polar analytes while a non-polar fiber coating will more effectively absorb non-polar analytes. Additionally, increasing the thickness/amount of the coating present on the fiber will result in more of the analyte being adsorbed by the coating [21]. The kinetics of the adsorption process can vary considerably. The rate of analyte adsorption is dependent on the adsorbent used, nature of analyte(s), thickness of the adsorbent film, amount of sample agitation, and temperature. Typical adsorption times vary from around 5 min to around 60 min [22,23].

The appropriate selection of a fiber coating is one of the most critical steps of SPME method development. The suitability of the given fiber coating for a specific analyte of interest is determined by the polarity of the coating and its selectivity toward the analytes of interest in contrast to the other matrix components. SMPE coatings consist of either a simple film or a mixture which forms a film with porous particles embedded in it. The presence of porous particles provide a much larger surface area which enables a greater amount of the analyte to be adsorbed. This type of coating is particularly useful for the adsorption of trace-level analytes in a sample. Commonly used SPME fibers coated with a simple film are PDMS (non-polar), PA (polar), and CAR (polar). Commonly used SPME fibers coated with a mixture of a film and porous particles are CAR/PDMS (bipolar), PDMS/DVB (bipolar), and DVB/CAR/PDMS (bipolar) [24].

## 2. Optimizing SMPE Analyte Extraction

### 2.1. Effect of Ionic Strength on Analyte Extraction from Samples

Extraction efficiency can also be significantly enhanced via the addition of a salt (usually NaCl) to the aqueous analyte sample. An increase in ionic strength makes hydrophobic compounds less soluble in the aqueous solution (salting-out effect) and also alters the nature of the phase boundary between the stationary phase and sample solution. This can result in more of the analyte being adsorbed by the fiber.

**Figure 2:**
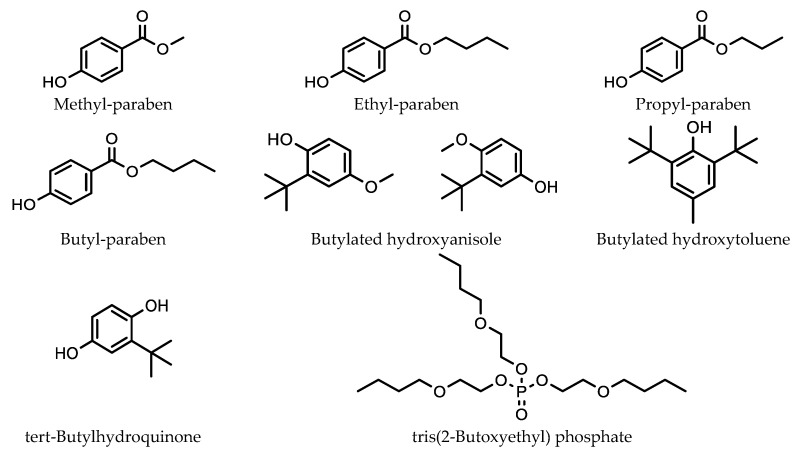
Preservatives and antioxidants used in food packaging investigated by Žnideršič et al.

For example, Žnideršič et al. used SPME to analyze compounds commonly found in food packaging (methyl-, ethyl-, propyl-, and butyl- paraben, and butylated hydroxyanisole, butylated hydroxytoluene, tert-butylhydroquinone, and tris(2-butoxyethyl) phosphate) (Figure 2) that can leach into alcoholic drinks and vinegars [25].

They investigated extraction efficiency at NaCl concentrations of 0, 50, 100, and 200 g/L. They found that, in most cases, increasing the analyte’s NaCl concentration, resulted in an overall increase in extraction efficiency. However, the increase observed varied considerably between the compounds investigated. For butylated hydroxyanisole, extraction efficiency increased by a factor of 3.5. There was an increase of 4 for butyl paraben, 6 for methyl paraben, 7.5 for propyl paraben, and 8 for ethyl paraben. However, for butylated hydroxytoluene, there was little change in extraction efficiency between 0 and 100 g/L NaCl, while at 200 g/L NaCl, there was in fact a decrease in extraction efficiency of 50%.

SPME has also been used to quantify off-flavors from corks (referred to as wine taint) used in wine bottles in order to assess wine quality. Jové et al. developed a method for the analysis of compounds in corks responsible for cork-tainted wine [26]. HS-SPME, with a DVB/CAR/PDMS fiber combined with GC-MS, was used for analysis. The compounds analyzed consisted of a number of chlorinated anisoles (2,4,6–trichloroanisole, 2,3,4,6-tetrachloroanisole, 2,4,6-tribromoanisole, and pentachloroanisole), pyrazines (2-isopropyl-3-methoxypyrazine, 2-methoxy-3,5-dimethylpyrazine, and 2-isobutyl-3-methoxypyrazine), and miscellaneous compounds, 2-methoxyphenol, 2-methylisoborneol (a monoterpene) and geosmin (a sesquiterpenoid). These compounds have a very low odor threshold of perception (around 5 to 25 ppt). They can leach out from the cork, and impart a woody, dank, acrid aroma and flavor to the wine. The addition of NaCl at a concentration of 300 g L^−1^ increased the extraction of chlorinated compounds by a factor of four. Extraction for the other compounds was even greater with an increase in extraction by a factor of 14.

**Figure 3:**
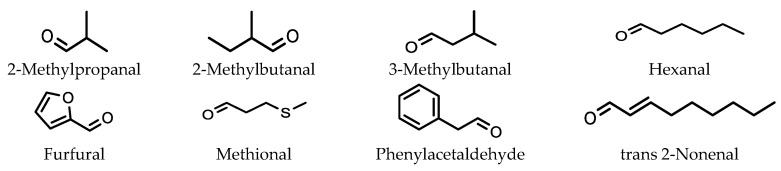
Aldehydes responsible for flavor in beer extracted using a PDMS/DVB SMPE fiber.

Filipowska et al. optimized extraction for a number of aldehydes from pale malts for analysis using HS-SPME [27]. These aldehydes are important compounds that affect the flavor of beer. The marker aldehyde compounds analyzed were 2-methylpropanal, 2-methylbutanal, 3-methylbutanal, hexanal, furfural, methional, phenylacetaldehyde and trans 2-nonenal (Figure 3). A 65 μm PDMS/DVB SMPE fiber was used for the extraction.

Extraction conditions that were investigated included the amount of the sample, extraction time, extraction temperature, and ionic strength. When 0.5 to 2.0 g of NaCl was added to the GC vial, no significant effect on the amount of aldehydes extracted was observed.

The type of salt used is also an important factor to consider in optimizing extraction efficiency. The effect of a number of salts/salt mixtures (NaCl, Na_2_SO_4_, (NH_4_)_2_SO_4_, and NaH_2_PO_4_) on the extraction of free fatty acids (C2-C10) in cheese and wine samples was investigated by Fiorini et al. [28]. The HS-SMPE analysis involved the use of a 50/30 μm DVB/CAR/PDMS fiber. Extraction was performed at 35 °C with constant stirring, with incubation and extraction times of 30 and 60 min, respectively. Extraction salts were compared to a saturated NaCl analyte solution. When using NaCl, there was an overall steady increase in extraction efficiency from C2 to C10. However, for C2–C6 the best analyte extraction was obtained using a (NH_4_)_2_SO_4_/NaH_2_PO_4_ mixture. Here, extraction efficiency increased from C2 to C6. However, from C8 to C10, extraction efficiency decreased.

### 2.2. Effect of Extraction Efficiency on pH

pH can also have a significant effect on extraction efficiency for acidic and basic analytes in a sample [29]. Weakly acidic and basic analytes will be more effectively extracted in their non-ionized (less polar) form by a SPME fiber. Therefore, to optimize extraction, lower pHs are favored for weakly acidic analytes, while higher pHs are favored for weakly basic analytes. If both weakly acid and basic analytes are present in a sample, a compromise needs to be made on sample pH in order to maximize extraction of all of the different types of analytes present.

**Figure 4:**
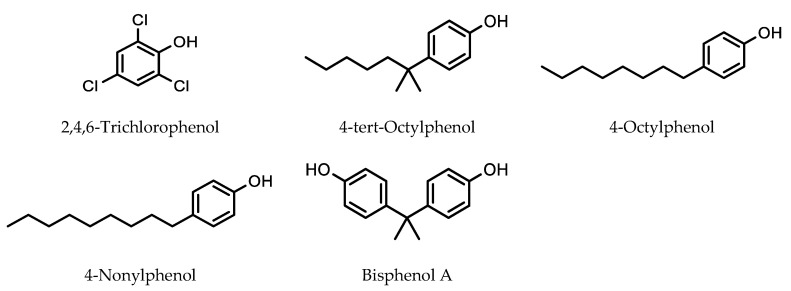
Endocrine-disrupting chemicals extracted using a PDMS/DVB SPME fiber.

Chammui used direct SPME to develop a method to analyze a number of endocrine-disrupting chemicals leaching from plastic baby milk bottles [30]. In method development, a 65 μm polydimethylsiloxane/divinylbenzene (PDMS/DVB)-coated fiber was chosen to be optimal for the extraction of endocrine-disrupting chemicals 2,4,6-trichlorophenol, 4-tert-octylphenol, 4-octylphenol, 4-nonylphenol, and bisphenol A (Figure 4). Extraction efficiency for bisphenol A was higher by a factor of around 1.6 at pH 2 compared to pH 8, while for 2,4,6-trichlorophenol it was higher by a factor of around 2. However, for 4-tert-octylphenol, 4-octylphenol and 4-nonylphenol, extraction efficiency was essentially unchanged over a pH range of 2–8.

## 3. Flavors and Aromas (Volatile Organic Compounds) in Foods Using HS-SPME

Food aroma analysis can be an extremely difficult task in food as it is complicated by a number of factors such as low concentrations, matrix effects, the presence of non-volatile components such as lipids, proteins and carbohydrates, complexities of food aromas and flavors, variations in analyte volatilities, and analyte instability [31]. Quantitative analysis of volatile and semi-volatile flavor-contributing chemicals in food products can be performed using SPME combined with GC-MS. Furthermore, it is rapid, less expensive, and offers analytical precision as good as or better than that of most sample preparation techniques widely used today for flavor analysis [10].

### 3.1. Volatile Organic Compounds in Fruit/Fruit Juice

Steingass et al. investigated the changes in the profile of volatile compounds as a function of aging in fresh pineapple juice under conditions of accelerated aging over a 16 week period at 25 °C [32]. For GC-MS analysis, samples were prepared using a 65 μm PDMS/DVB SPME fiber, with an extraction time of 60 min at 40 °C. They proposed that Maillard reaction products, 4-hydroxy-2,5-dimethyl-3(2H) furanone and 4-methoxy-2,5-dimethyl-3(2H)-furanone could be used as marker compounds to monitor the aging profile of pineapple juice.

Facundo et al. used headspace solid-phase microextraction (HS-SMPE) to extract aroma compounds from whole bananas and banana pulp [33]. A 50/30 μm DVB/CAR/PDMS SMPE fiber was used together with a response surface methodology to optimize the extraction of these compounds. They found that the extraction temperature did not significantly affect extraction so it was set at 25 °C for the optimization process. Fiber exposure and headspace equilibrium times were modeled to determine optimum extraction conditions (whole bananas: 140 min equilibrium and 120 min exposure; banana pulp: 15 min equilibrium and 60 min exposure). Whole bananas and banana pulp had similar volatile profiles mainly consisting of esters. Aromas from green fruit pulp mainly contained acids and aldehydes while in those from whole fruit, terpenes were mainly observed.

Recently, Shimizu et al. reported on the use of HS-SPME and GC-MS to profile mango cultivars using principle component analysis and the volatile organic profile data from each cultivar [34]. Here, 17 different mango cultivars were analyzed with 59 volatile organic compounds detected. These mainly consisted of monoterpenes (e.g., α-pinene, α-limonene, camphene, γ-terpinene, etc.) and sesquiterpenes (e.g., germacrene-D, *α*-copaene, valencene, alloaromadendrene, etc.), together with a smaller number of other types of compounds. Extraction was performed using 50/30 μm of DVB/CAR/PDMS SPME fiber at 50 °C for 60 s, stirring at a rate of 600 rpm. A desorption time of 60 s and a desorption temperature of 230 °C were used. Principle component analysis using the volatile compound analysis data divided the cultivars into four distinct groups.

**Figure 5:**
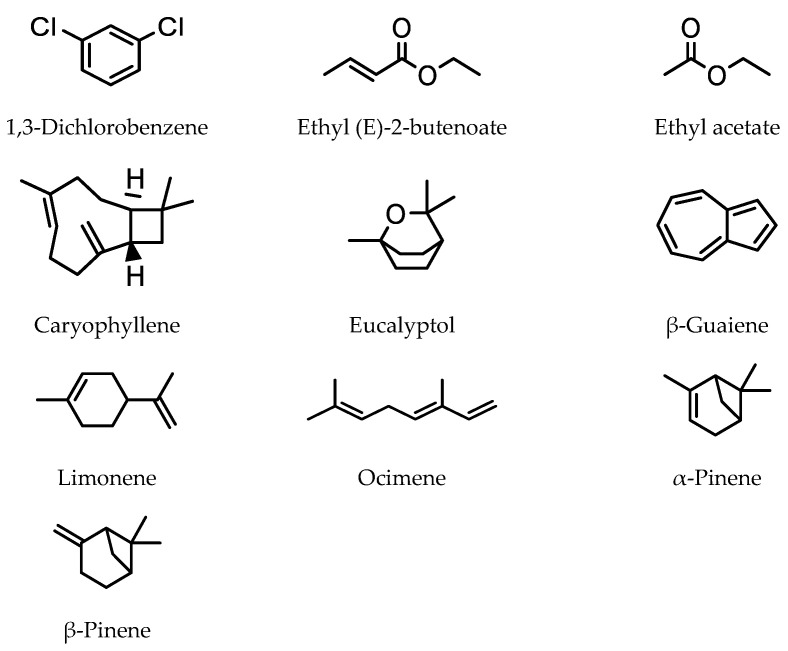
Main volatile compounds extracted from *Myrciaria jaboticaba* fruit.

Sanabria et al. developed a HS-SMPE GC-MS method to detect volatile compounds in jabuticaba fruit (*Myrciaria jabuticaba*), a small grape-like fruit native to Brazil [35]. In total, 71 compound were extracted and identified. Both a 85 μm CAR/PDMS and a 50/30 μm DVB/CAR/PDMS SMPE fiber were used for the extraction of volatile organic compounds. The 85 μm CAR/PDMS SMPE fiber provided better extraction, with a greater number of volatile organic compounds being detected. A response surface methodology was used to optimize extraction conditions. The most effective extraction occurred using an extraction time of 30 min at 42 °C, with 5% NaCl. The main compounds extracted were 1,3-dichlorobenzene 9.7%, esters ethyl (E)-2-butenoate 2.5% and ethyl acetate 10.7%, and terpenoids caryophyllene 5%, eucalyptol 5.8%, β-guaiene 2.9%, limonene 17.7%, ocimene 3.6%, α-pinene 2.2%, and β-pinene 3.0% (Figure 5).

The two volatile compounds with the highest concentrations were limonene 17.7% and ethyl acetate 10.7%.

### 3.2. Volatile Organic Compound Profile in Oranges Correlated with Farming Practice

Cuevas et al. investigated the use of HS-SPME GC-MS in the analysis of aroma compounds in the fresh fruit of two orange cultivars, Navalina and Salustiana (*Citrus sinensis* L. Osbeck), grown via conventional methods and organically [36]. Their aim was to use the aroma profiles of fresh fruit pulp to determine if orange samples were grown via conventional methods or organically. A number of volatile aldehydes, ketones, alcohols, terpenoids, esters, and also 2-ethyl furan were assessed. Analysis consisted of headspace sampling using a 50/30 μm CAR/PDMS/DVB fiber (StableFlex/SS) (Supelco). Both incubation and extraction were carried out at 60 °C. The incubation time was 10 min and the extraction time was 30 min, with the sample being continuously agitated at 500 rpm. The sample desorption time was 15 min at a temperature of 250 °C. They found that conventionally farmed oranges had a higher concentration of esters, while organically farmed oranges had a higher concentration of neryl acetate, geranyl acetate, and some monoterpenes. Applying partial least squares discriminate analysis to the data enabled them to successfully predict what farming method was used in the production of the orange samples.

### 3.3. Volatile Organic Compounds and Wine Quality

HS-SPME combined with GC-MS has been widely used to determine the presence and concentration of volatile organic compounds in wine [7,37,38]. Many of these compounds are important in assessing wine quality, so it is important to have effective and reliable analytical methods for determining the presence of these compounds. Compounds responsible for aroma and taste can be classified into the following groups: aliphatic alcohols, benzenoids, carboxylic acids, sesquiterpenoids, and terpenes. Recently, Rossi et al. used HS-SPME with GC-MS to determine the volatile organic compound profile in two white wines (Trebbiano d’Abruzzo and Pecorino) from Abruzzo, Italy [39]. In their work, they used a response surface methodology in order to optimize the sensitivity of their method. The parameters optimized were the type of fiber used (either PDMS, CW/DVB, or PDMS/CAR/DVB), fiber exposure time, temperature, and NaCl concentration. Two different compounds, typical of those present in the wine volatile organic profile (ethyl decanoate (non-polar) and 3-methyl-1-butanol (polar)), were used as analytes in order to simplify the optimization process. The PDMS/CAR/DVB SMPE fiber extracted the largest number (around 70) of volatile organic compounds. The optimization process showed that the use of 10% NaCl with an extraction temperature of 50 °C or 30% NaCl with an extraction temperature of 30 °C, both resulted in the maximum extraction of volatile components. However, fewer volatile esters were preferentially extracted with 10% NaCl at 50 °C while more volatile esters were preferentially extracted with 30% NaCl at 30 °C.

Rocha et al. used HS-SMPE to investigate the effect of the volatile wine composition on the response factor measured for a given volatile organic compound [40]. In order to simplify the work, nine volatile compounds were tested (ethyl hexanoate, ethyl octanoate, ethyl decanoate, geraniol, hexanol, linalool, 3-methyl-1-butanol, nerol, and 2-phenylethanol). A 85 μm PA SMPE fiber was used for all analyses. Method optimization involved investigating the sample volume, fiber exposure time, extraction temperature and NaCl concentration. There was an increase in the peak area when the sample was stirred at 1000 rpm from 1.4 times for ethyl decanoate to 3.1 for linalool. When 200 g/L of NaCl was used, an increase of 1.4 times for ethyl decanoate to 6.4 for linalool was observed. However, extraction under increasing temperatures, 25, 30 and 40 °C, did not significantly change the amount of volatile compounds extracted, so 25 °C was used in all subsequent measurements. Changing the sample volume from 25, to 30 and 40 mL did change the amount of terpenes and alcohols adsorbed. A sample volume of 30 mL was chosen, as the amount of terpenoids adsorbed did increase when the sample volume increased from 20 mL to 30 mL. It was observed that significantly increasing the concentration of a particular volatile organic compound resulted in a decrease in adsorption of the other volatile organic compounds present. In particular, compounds with high relative response factors were less affected by matrix effects in the sample.

## 4. HS-SMPE Arrow Applications in Food Analysis

**Figure 6:**
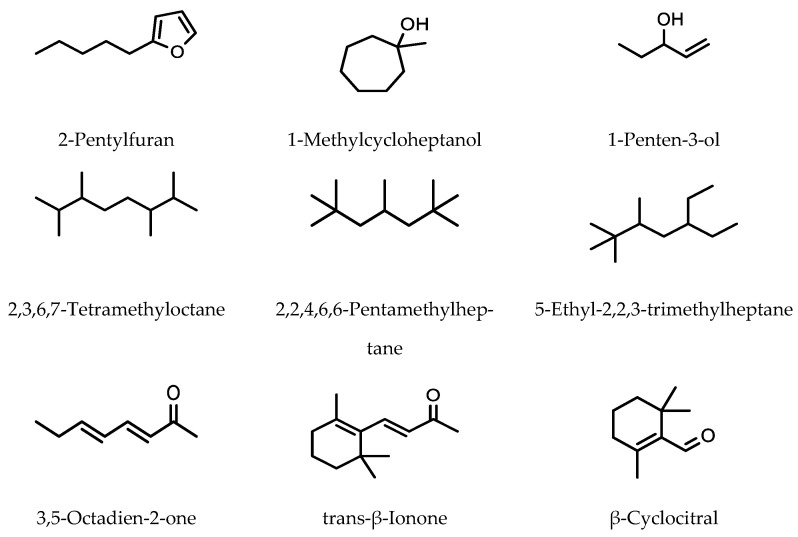
Major volatile organic compounds identified in *Cirsium setidens* Nakai using carbon wide-range/PDMS SMPE Arrow.

The use of a SMPE Arrow for sample preparation in analysis enables much larger amounts of analytes to be collected from the sample matrix. However, there has not been a great deal of research investigating volatile components in food using SMPE Arrow. Kim et al. used SPME Arrow with GC-MS to analyze the volatile components in *Cirsium setidens* Nakai, a Korean herb that is used to flavor steamed rice and is used as a traditional medicine [41]. In developing their method, they compared a four different SMPE Arrows for analyte extraction: (1) a PDMS SMPE Arrow, (2) a PA SMPE Arrow, (3) a DVB/PDMS SMPE Arrow, and (4) a carbon wide-range/PDMS SMPE Arrow. The most effective extraction of volatile components (aldehydes, aliphatic hydrocarbons, furans, nitro and nitrate compounds, and pyrazines and terpenes) was performed using the carbon wide-range/PDMS SMPE Arrow with 58 volatile compounds being identified (Figure 6).

Compared to the other SMPE Arrows, the results obtained using the carbon wide-range/PDMS SMPE Arrow had the lowest relative standard deviation for the analytes measured. The extraction conditions were as follows. Samples were equilibrated for 10 min. SMPE Arrow was then placed into the headspace for 30 min, after which desorption occurred at 250 °C for 5 min.

Nam et al. compared the headspace analysis of volatiles in brown rice vinegar using both SPME and a SPME Arrow [42]. They compared four different types of SPME fiber and found that a CAR/PDMS SMPE fiber resulted in the best extraction of volatile organic compounds. GC-MS headspace analyses using a conventional CAR/PDMS SMPE and a CAR/PDMS SMPE Arrow were then compared. Samples were equilibrated at 50 °C for 30 min for each SPME system. Overall, there was a 1.3- to 2-fold increase in sensitivity, together with lower uncertainty in concentrations determined using the CAR/PDMS SMPE Arrow. Furthermore, more compounds were extracted using the CAR/PDMS SMPE Arrow compared to the conventional CAR/PDMS SMPE fiber (i.e., 12 versus 7 volatile organic compounds).

More recently, Huang et al. used a SPME Arrow to determine furan content in 31 different food products (oats, malt extract, rice cereal, donut, muffin, grilled beef kebab, chicken nuggets, whole milk, etc.) using HS GC-MS [43]. Furans are an undesirable contaminant arising from heat-processed foods and are considered possible carcinogens [44]. GC-MS headspace analysis was performed using a CAR/PDMS cellulose SPME Arrow with an extraction temperature of 30 °C. The method enabled ng/g concentrations of furans to be measured. The results showed that the total furan concentration was highest in brewed coffee (around 35,000 ng/g), and that it was the lowest in potato chips and cookies (0.57–1.48 ng/g).

## 5. Applications of SPME and HPLC in Agricultural Products

Desorption using SMPE-GC occurs under high temperatures, via the placement of the fiber directly into the gaseous mobile phase in the GC injection port, while HPLC desorption relies on the ability of the mobile phase to desorb the sample. If sample desorption does not occur quickly, undesirable peak broadening in the HPLC column will occur. This can be overcome by dipping the fiber in a small volume of the mobile phase or another suitable solvent until effective desorption takes place (static desorption). After desorption, the mobile phase/solvent sample is then injected into the HPLC column. However, the lifetime of SMPE fibers is often reduced when used with HPLC, due to swelling and break down of fiber coatings when exposed to mobile phase solvents [45]. The combination of SMPE with HPLC is particularly useful where analytes under investigation are non-volatile and/or break down under the elevated temperatures used in GC analysis [46]. This method has been widely used in the analysis of a wide range of pesticides [47].

### 5.1. Analysis of Pesticides, Herbicides and Environmental Pollutants in Agricultural Products Using SMPE and HPLC

**Figure 7:**
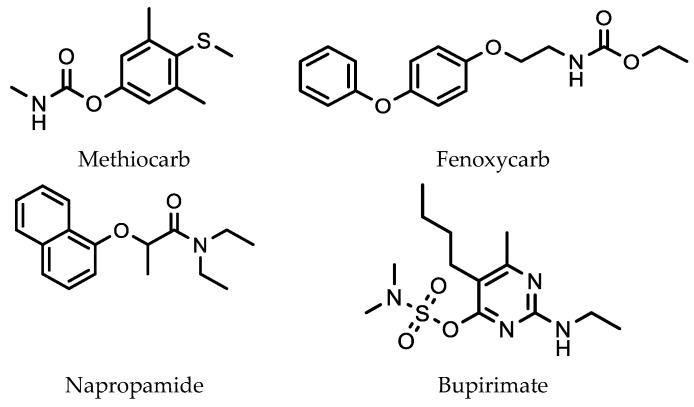
Pesticide residues on strawberries extracted using a 60 μm PDMS/DVB SPME fiber.

An early application of SMPE with HPLC was reported by Wang et al. for the analysis of four pesticides (Figure 7) used on strawberries [48].

In developing their method, four SPME fibers were investigated. These consisted of a 100 μm PDMS, 60 μm PDMS/DVB, 75 μm CAR-PDMS, and a 50 μm CW/TPR fiber. Each fiber was subjected to an extraction time of 45 min with the solution stirred at 1000 rpm. A desorption time of 1 min in a small volume of the mobile phase (acetonitrile (55% *v*/*v*)–water (45% *v*/*v*)) was then used. The best SPME fiber for the extraction of these pesticides was the 60 μm PDMS/DVB fiber. The use of a SPME fiber resulted in a sensitivity of around five times greater when compared to that under the direct injection of the pesticide mixture into the HPLC.

Diethystilbestrol (DES) is a synthetic non-steroidal estrogen that was widely used in the past to treat a number of diseases and also to promote growth in animals for improved food production [49]. However, DES and its metabolites do not break down easily in the environment and they have been found to be carcinogenic. Even though DES is no longer used as a medication and it is use in farming has been banned in many counties, it persists in the environment for a long period of time. Therefore, it is important to carefully monitor its presence both in the environment and in food products. Yang et al. used sol–gel technology to fill wall pores of a carbon nanotube-reinforced hollow SPME fiber (CNTs-HF-SPME) with multi-walled carbon nanotubes (MWCNTs), in order to extract DES from milk products for HPLC determination [50]. The MWCNTs in the wall pores of the hollow fiber were found to selectively extract DES from milk products. Hollow fiber cartridges have the advantages of high surface area and good semi-permeability. They can limit large molecules such as proteins from entering small pores. Since MWCNTs are hydrophobic, 1-octanol was used as a wetting agent to promote the adsorption of DES from milk samples. Method optimization resulted in an extraction time of 30 min, with stirring (800 rpm) at 60 °C and a pH of 3.0. The desorption of DES from the fiber involved the use of methanol and ultrasonication over a period of 10 min. A wide range of linearity between 24 and 960 μg/L, together with a low detection limit of 5.1 μg/L, was obtained using this method.

A major limitation of SPME Is Its inability to extract highly polar compounds from a sample. This was an issue for Melo et al. when developing a SPME extraction method combined with HPLC and a diode array detector (DAD) to analyze three insecticides and seven fungicides commonly used in agriculture [51]. Method development involved comparing the extraction performance of four different SPME fibers: PDMS, PDMS/DVB, CW/TPR, and PA. The CW/TPR polar fiber was selected as the most appropriate for extraction. Good extraction was observed for seven of the fungicides that had a log *P* of >2.5. However, here was little extraction observed for the more polar compounds, primicarb (log *P* = 1.7) and metalaxyl (log *P* = 1.65), while no extraction for acetamiprid (log *P* = 0.8) was observed. The experimental variables pH, NaCl concentration, and extraction time were optimized for analyte extraction using a surface response methodology. This resulted in the optimal extraction of samples at pH 8, 18% *w/v* NaCl, and a 30 min extraction time (with stirring at 1000 rpm at room temperature). Fiber desorption took place for 10 min in a 60 μL aliquot of a 90% *v/v* aqueous methanol solution. The solution was then injected into the HPLC column for analysis. The developed method was used to determine fungicide concentrations in a crop of lettuce. Fungicides azoxystrobin, cyprodinil, fenehexamid, fludioxonil, folpet, iprodione, and tolyfluanid were able to be determined when over a concentration range from 0.8 to 25.6 mg/kg. The method showed good sensitivity, with the lower limit being well below the acceptable residue concentrations for these fungicides.

**Figure 8:**
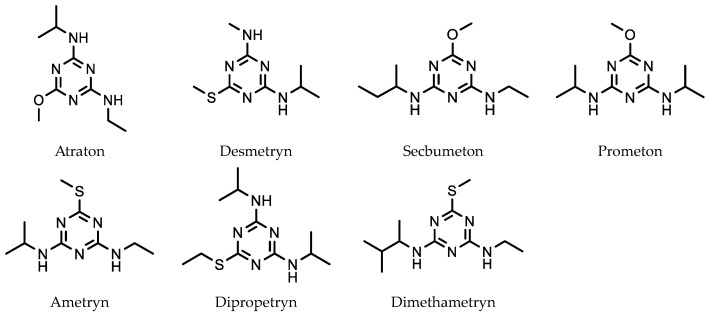
Triazine herbicides extracted using a chromium terephthalate porous solid chitosan absorbent.

Triazine herbicides are widely used on crops to control weeds. Recently, Jiang et al. developed a SPME HPLC-MS method for the analysis of seven triazine herbicides (Figure 8) [52].

This was based on a novel absorbent, consisting of a thin film of a mixture of chromium terephthalate porous solid (MIL-101(Cr)) [53] and chitosan, coated on a cellulose filter paper substrate. Discs with a diameter of 1.5 cm were prepared for herbicide extraction. This type of SPME is referred to as thin-film microextraction (TFME). The advantage of TFME over extraction using a traditional SMPE fiber is that it provides both a thin film together with a relatively large surface area. This enables the good adsorption of an analyte with relatively low extraction and desorption times. The optimal extraction and desorption times for the method were 15 min each. A 5 mL analyte sample was used for the extraction while desorption involved the disk being placed in a tube with 2.5 mL of acetonitrile, which was then evaporated to dryness. The dried extracted sample was then redissolved in 1 mL of methanol before being injected into the HPLC column. A LOD of 0.022 ng/L compared favorably with (or was lower than) the LODs for a number of other different extraction methods. The adsorption of the triazine herbicides from the water sample was thought to be due to π–π interactions between the terphthalic acid groups in the adsorbent layer and the heterocyclic rings in the triazine herbicide molecules.

### 5.2. Analysis of Phenolic Acids in Orange Juice Using SMPE and HPLC

**Figure 9:**
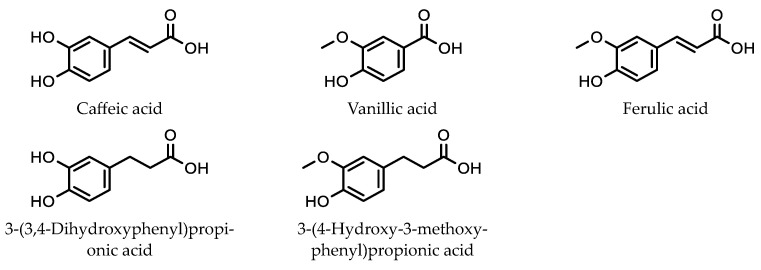
Extraction of phenolic acids using an adsorbent of poly (1-allyl-3-methylimidazolium bis [(trifluoromethyl) sulfonyl] imide-co-ethylene dimethacrylate) polymer embedded with Fe_3_O_4_ nanoparticles.

Chen et al. used in-tube solid-phase microextraction for the analysis of five phenolic acids (Figure 9) present in orange juice samples. This involved using in-tube solid-phase microextraction, with subsequent analysis using a HPLC equipped with a DAD [54]. The adsorbent coating on the inside of the capillary consisted of a poly (1-allyl-3-methylimidazolium bis [(trifluoromethyl) sulfonyl] imide-co-ethylene dimethacrylate) polymer embedded with Fe_3_O_4_ nanoparticles. The extraction of the phenolic acids from the juice sample was enhanced via the use of a magnetic field around the capillary.

Juice samples were first centrifuged, filtered, diluted, and adjusted to pH 6, before being pumped through the capillary. The adsorption phase consisted of pumping a 4.5 mL prepared orange juice sample into the capillary at 100 μL/min. The desorption of phenolic acids involved pumping 120 μL of methanol though the column at 40 μL/min. The methanol sample was then injected into a HPLC for analysis. The method showed good linearity (0.10–300 μg/L) and a good limit of quantitation (0.040–0.19 μg/L) for the five phenolic acids investigated.

## 6. Conclusions

SPME combined with either GC-MS or HPLC has a number of advantages over traditional methods for extracting impurities. It is much cheaper, and has a faster sampling time, with only small amounts of solvent being used. Importantly, minimizing solvent use reduces the impact of waste organic solvents on the environment. For this reason, many SPME analysis methods have been developed covering a wide range of different applications for food, plant, pesticide, herbicide, and fungicide analysis, for example, the analysis of different fruits, herbs, fruit juices, coffee, etc. However, more ambitious analytical limits are continuously being reported and emergent sorbents, based on carbon nanotubes and magnetic nanoparticles, will improve the sensitivity of this extraction method [55].

## Data Availability

Not applicable.
